# Learning About Your Mental Health From Your Playlist? Investigating the Correlation Between Music Preference and Mental Health of College Students

**DOI:** 10.3389/fpsyg.2022.824789

**Published:** 2022-04-22

**Authors:** Kun Wang, Sunyu Gao, Jianhao Huang

**Affiliations:** ^1^Graduate University of Mongolia, Ulan Bator, Mongolia; ^2^Southern University of Science and Technology, Shenzhen, China; ^3^Dhurakij Pundit University, Bangkok, Thailand

**Keywords:** music preference, college students, correlational study, common method variance, mental health

## Abstract

The present study explored the correlation between music preference and mental health of college students to make an empirical contribution to research in this field. The self-reported music preference scale and positive mental health scale of college students were adopted to conduct a questionnaire survey in college students. Common method variance was conducted to test any serious common method bias problem. No serious common method bias problem was observed. The results showed that college students’ preference for pop music, Western classical music, and Chinese traditional music has a significant and positive correlation with their mental health. Furthermore, college students’ preference for heavy music has a significant and inverse correlation with their mental health. This research presents a correlational study; therefore, no causality can be inferred.

## Introduction

The World Health Organization statement “there is no real health without mental health” has long become a global principle (WHO, 2013). Mental health of college students and the measures that should be adopted by universities to deal with this problem have become a major concern (Castillo and Schwartz, 2013). Some researchers have reported that the mental health problems of college students are becoming increasingly complex and serious (Pledge et al., 1998; Benton et al., 2003). The increased prevalence of symptoms of depression, anxiety, eating disorders, and other mental diseases among college points toward a mental health crisis. Therefore, conducting active surveys and seeking possible solutions to meet the needs of college students are imperative (Lattie et al., 2019). Accordingly, the key factors influencing college students’ mental health should be explored to provide an empirical basis for improving their mental health.

Music can help college students adjust their mental state, release inner stress and pain, and express happiness, thereby serving as a means of decompression (Huang et al., 2020). It can also evoke inner feelings and guide emotions, which are the key factors influencing individuals’ cognition, decisions, and actions (Koelsch, 2015). Researchers have been paying increasing attention to the students’ music preference (Schwartz and Fouts, 2003; Ballmann, 2021). Earlier, some researchers defined music preference as people’s preference for certain music in the face of two or more choices (Rentfrow and Gosling, 2003). In addition, some researchers argued that music preference is a decision on the overall music stimulation, which is made by individuals after listening to the whole piece of music and may continue to occur long after listening to the music (Brattico and Pearce, 2013). This study adopted the latter view and defined music preference as the degree of preference of college students for a specific music pattern after the overall music stimulation. Schwartz and Fouts (2003) classified music preference into three types, namely heavy music, light music, and compromised music. Liu (2020), a Chinese researcher, classified music preference into classical music, pop music, and Chinese folk music. Rentfrow and Gosling (2003) categorized music preference into five types, namely Chinese traditional music, percussion/Hip-hop music, pop music, classical music, and opera. In the present study, music preference was classified into pop music, Western classical music, Chinese traditional music, and heavy music because the studied cohort in this study was Chinese college students. The study explored the music preference of Chinese college students in the context of Chinese culture.

Some researchers have asserted that people exposed to positive music hold a positive attitude and exhibit less negative experience, thus presenting a better mental state (Yuan, 2020). In other words, the mental state and mood of individuals who prefer to listen to positive music is better. However, these individuals may feel sad or depressed when they listen to the music of not their choice (Rentfrow, 2012). Janata et al. (2012) reported that the correlation of music preference with relieving stress and regulating psychology varies among individuals. Empirical studies have found that music preference has a significant correlation with mental health (Carlson et al., 2015). Music can invoke various emotions, which are reflected in individual physiological signals and the correlation with mental health (Rahman et al., 2021). This study inferred that college students’ music preference may exhibit a correlation with their mental health.

Most studies on the students’ music preference have been conducted in Western countries (Carlson et al., 2015). However, the Western culture differs greatly from the Chinese culture (Rentfrow and Gosling, 2003). Therefore, this study considered Chinese college students as the sample and adopted the Chinese local music preference scale to explore the correlation between music preference and mental health of Chinese college students, making an empirical contribution to research on music preference and mental health.

## Theoretical Background and Hypotheses

According to Stimulus-Organism-Response theory, individuals’ response is triggered by their internal emotional state after being stimulated (Mehrabian and Russell, 1974). The theory holds that most changes in the environment act as a stimulus for an individual, which results in the transmission of information to the individual’s nervous system, leading to reactions to muscles or psychology (Bergius, 1994). Some researchers adopted this theory to explore the relation between music and individual psychology (Juslin and Västfjäll, 2008), whereas other researchers adopted the theory to explore the relation between music environment and individual behavior intention (Zhuang et al., 2020). Music is one of those stimuli, and listeners may exhibit preference for music when the stimulation of music is consistent with their mental state (Droe, 2006). In addition, music preference has an correlation between individuals’ emotions and mental health. Therefore, we adopted this theory to determine the correlation between music preference and mental health of college students.

### Pop Music

Pop music involves music works with popular contents and sincere emotions that can be accepted, preferred, appreciated, and sung by listeners (Shuker, 2013). Pop music style can help listeners express their values and abilities, which in turn helps them gain recognition and make new friends, thus resulting in positive psychology (Schäfer et al., 2012). Pop music can also help teenagers express their values, aspirations, beliefs, and views on the world, as well as explore and express their identities and increase understandings of their thoughts and feelings, which play a crucial role in their development (North and Hargreaves, 1999; Rentfrow and Gosling, 2006). This music form is also associated with the empathy of teenagers, can enhance interpersonal relationships, and explore others’ personalities. It serves a medium to facilitate communication and common activities, which are conducive to individuals’ physical and mental health (Lull, 1987; Rentfrow and Gosling, 2006). Therefore, we proposed H1: College students’ preference of pop music is significantly and positively correlated to their mental health.

### Western Classical Music

Western classical music has been reported to exert a positive correlation between individuals’ mental health and daily behavior, as well as reduce anxiety and alleviate depression in patients with psychiatric disorders such as schizophrenia (Harmat et al., 2008; Rahman et al., 2021). Moreover, classical music has been shown to exert a positive correlation between individuals’ mental health and daily behavior by controlling their attention level (Baldwin and Lewis, 2017; Rahman et al., 2021). Some researchers have proved that classical music can exert a strong decompression effect, helping listeners in relieving tension and relaxing (Burns et al., 2002). Individuals may seek excitement in music that is similar to their emotions. A study indicated that listening to classical music increases the sense of relaxation and is conducive to the reduction of negative emotions, which is beneficial to people’s mental health (Rea et al., 2012). Therefore, we proposed H2: College students’ preference of Western classical music is significantly and positively correlated to their mental health.

### Chinese Traditional Music

Chinese traditional music generally refers to various forms of Chinese music that has been passed on through generations, with national characteristics and creativity (Yung, 2019). Previous studies have shown that traditional music can help young people improve their sense of identity, motivate themselves, awaken their ability to express values, and obtain opportunities to know other people (Schäfer and Sedlmeier, 2009; Schubert et al., 2020). Some studies have indicated that Chinese traditional music can achieving the goal of aesthetic education, implying that the increased positive emotions in Chinese traditional music can gradually convert even the negative and sad experience into positive and lively experience, thereby providing a physiological feedback to individuals (Liu et al., 2021). Listening to Chinese traditional music can trigger the brain to produce endorphins to regulate unpleasant feelings and emotions, thus improving mental health (Lin et al., 2019). According to aforementioned studies, Chinese traditional music is closely related to the positive emotions of college students. Therefore, we proposed H3: College students’ preference of Chinese traditional music is significantly and positively correlated to their mental health.

### Heavy Music

In general, heavy music comprises all music styles characterized by metal music, including rock music, heavy metal music, and rap music, which usually produce loud and fast melodies to express intense emotions such as madness and roughness (Larson, 1995). Views on the relation between heavy music and mental health in previous studies have been inconsistent and contradictory. Some researchers have argued that individuals can release their emotions, anxiety, and anger after listening to heavy music (Martin et al., 1993). In addition, this music form can help listeners believe that they are not alone emotionally by helping them find solace and increase their sense of connection (Arnett, 1991). Conversely, other researchers have asserted that people who prefer heavy music experience more adverse outcomes such as depression, anxiety, and drug addiction, which can deteriorate their mental health (Miller and Quigley, 2012; Shafron and Karno, 2013; Monteiro et al., 2021). A few music fans reported worse experience after listening to heavy music and were prone to suicidal thoughts or self-destructive behaviors due to negative emotions produced by lyrics or theme artistic conception of the music (Miranda et al., 2012). Therefore, the contradictory and inconsistent research results warrant empirical research for deeper exploration. In the context of Chinese culture, domestic studies have found an association between heavy music and anxiety of Chinese college students. Students listening to heavy music are prone to be angry, irritated, and even self-doubted and depressed, which is not conducive to their mental health (Xu et al., 2010). Because of cultural differences between China and the West, Chinese college students’ preference for heavy music and their mental health may exhibit a inverse correlation. Therefore, we proposed H4: College students’ preference of heavy music is significantly and inversely correlated to their mental health.

## Materials and Methods

### Sample and Procedure

The scales of music preference of college students and positive mental health were adopted to conduct a questionnaire survey among college students in Shenzhen, Guangdong, China. A total of 139 students were selected through convenience sampling. The reliability and exploratory factor of the scales were analyzed using the pilot test data. Then, the data were collected from the students of three education reform pilot universities in Shenzhen, and all students participated voluntarily and anonymously. A total of 390 questionnaires were distributed, and 380 valid samples were obtained after removing 10 invalid samples, with a recovery rate of 97.4%, which met the sampling standard. Of the total, 198 participants were women and 182 participants were men. Additionally, the studied cohort comprised 81 freshmen, 116 sophomores, 118 junior grade students, and 65 senior grade students. The common method variance and correlation analysis were conducted on formal test data.

### Measurement

#### Music Preference Scale of College Students

To understand the music preference of Chinese college students, we first interviewed 40 college students in Shenzhen regarding the types of music they enjoy listening in daily life by using the scale developed by Liu and Wu (2018) and Larson (1995). We categorized the music preference scale into four dimensions in accordance with the interview results: (1) Pop music (Chinese mainland pop music, Hong Kong, Macao and Taiwan pop music, and Western pop music); (2) Western classical music (chamber music, symphony, and foreign piano works); (3) Chinese traditional music (Chinese folk songs, Chinese folk music, Chinese traditional opera and folk art); and (4) Heavy music (rock music, metal music, and rap music). The scale includes a total of 12 items, which are scored using the Likert’s five-point scoring method. The scores 1, 2, 3, 4, and 5 on the scale denote very dislike, dislike, between like and dislike, like, and very like, respectively.

#### Positive Mental Health Scale

The positive mental health scale developed by Lukat et al. (2016) was adopted, which comprises nine items and one dimension. The scale was scored using the Likert’s five-point scoring method, with 1 = srongly disagree; 2 = disagree; 3 = between disagree and agree; 4 = agree; and 5 = strongly agree.

#### Exploratory Factor Analysis

As shown in Table 1, the pilot test data was adopted to conduct an Exploratory Factor Analysis (EFA). According to the results, Kaiser-Meyer-Olkin (KMO) Test = 816, and the significance of Bartlett’s Test of Sphericity *p* < 0.001. When KMO Test is greater than 0.8, and the significance of Bartlett’s Test of Sphericity *p* < 0.05, the data are suitable for EFA (Kaiser and Rice, 1974). Therefore, the varimax rotation was adopted to conduct the EFA on the music preference scale. The rotated component matrix showed that four factors had eigenvalues >1, and their factor loadings ranged from 0.791 to 0.881, meeting the criterion of factor loading greater than 0.3 (Zaltman and Burger, 1975). The explained variance ratio of pop music, Western classical music, Chinese traditional music, and heavy music were 11.473, 7.207, 5.170, and 8.686%, respectively, and the cumulative explained variance ratio was 85.000%.

**TABLE 1:** Summary of the exploratory factor analysis on music preference scale of college students.

Items	Factor loading			
	
	Pop music	Western classical music	Chinese traditional music	Heavy music
**Pop music (The explained variance ratio: 21.435%)**				
1. Do you like Chinese mainland pop music?	0.885			
2. Do you like Hong Kong, Macao and Taiwan pop music?	0.892			
3. Do you like Western pop music?	0.894			
**Western classical music (The explained variance ratio: 12.989%)**				
4. Do you like chamber music?		0.873		
5. Do you like symphony?		0.877		
6. Do you like foreign piano works?		0.910		
**Chinese traditional music (The explained variance ratio: 11.504%)**				
7. Do you like Chinese folk songs?			0.845	
8. Do you like Chinese folk music?			0.891	
9. Do you like Chinese traditional opera and folk arts?			0.848	
**Heavy music (The explained variance ratio: 39.072%)**				
10. Do you like metal music?				0.935
11. Do you like rap music?				0.930
12. Do you like rock music?				0.935
The cumulative explained variance ratio = 85.000%				

As shown in Table 2, the pilot test data was adopted to conduct the EFA. The results showed that KMO Test = 965, and the significance of Bartlett’s Test of Sphericity *p* < 0.001, indicating the suitability of data for EFA (Kaiser and Rice, 1974). Therefore, the varimax rotation was adopted to conduct the EFA on the mental health scale. The rotated component matrix showed that one factor had eigenvalues >1, and its factor loading ranged from 0.841 to 0.885, meeting the criterion of factor loading greater than 0.3 (Zaltman and Burger, 1975). The cumulative explained variance ratio was 48.741%.

**TABLE 2:** Summary of exploratory factor analysis on positive mental health scale.

Items	Factor loading
1. I am always in a relaxed and cheerful mood.	0.848
2. I enjoy my life very much.	0.855
3. In a word, I am satisfied with my life.	0.881
4. In general, I am confident.	0.862
5. I can meet my needs well.	0.877
6. I am in good physical and mental condition.	0.853
7. I believe that I can handle life and the difficulties in life well.	0.854
8. Most of the things I do can bring me happiness.	0.885
9. I am a calm and balanced person.	0.841
The cumulative explained variance ratio = 74.287%	

#### Reliability Analysis

The pilot test data was adopted to conduct the reliability analysis on both music preference scale of college students and positive mental health scale. The reliability analysis of each dimension of the music preference scale indicated that the Cronbach’s α of pop music, Western classical music, Chinese traditional music, and heavy music was 0.931, 0.898, 0.892, and 0.836, respectively, indicating good reliability of all dimensions. Cronbach’s α of the positive mental health scale was 0.956, which indicated good reliability of the scale. The total Cronbach’s α of the questionnaire was 0.926, which indicated that the questionnaire also had good reliability.

### Data Analysis

AMOS version 22.0 was adopted to conduct a confirmatory factor analysis (CFA) on formal questionnaire results. In addition, the common method deviation test was conducted on the questionnaire by using SPSS version 22.0. The correlation between college students’ music preference and their mental health was determined using the Pearson correlation analysis.

## Results

### Confirmatory Factor Analysis and Reliability Analysis

Confirmatory factor analysis was conducted on formal questionnaire results of the music preference scale, and the results showed that χ^2^/df = 1.283, RMR = 0.022, GFI = 0.974, AGFI = 0.957, NFI = 0.982, IFI = 0.996, TLI = 0.994, CFI = 0.996, and RMSEA = 0.027, indicating that the measurement model fitted well with observations (McDonald and Ho, 2002). The factor loading ranged from.822 to.918, which is consistent with the standard. The CR of pop music, Western classical music, Chinese traditional music, and heavy music were.919,0.910,0.884, and.929, respectively, all of which are greater than the reference value of 0.6, meeting the standard. The AVE of pop music, Western classical music, Chinese traditional music, and heavy music were.791,0.772,0.718, and.814, respectively, which is consistent with the standard and indicates a good convergent validity. Cronbach’s α of pop music, Western classical music, Chinese traditional music, and heavy music were.918,0.909,0.883, and.929, respectively, indicating good reliability.

In addition, the CFA was conducted on formal questionnaire results of the mental health scale, and the results showed that χ^2^/df = 1.774, RMR = 0.015, GFI = 0.973, AGFI = 0.955, NFI = 0.985, IFI = 0.993, TLI = 0.991, CFI = 0.993, and RMSEA = 0.045, indicating that the measurement model fitted well with observations (McDonald and Ho, 2002). The factor loading ranged from 0.817 to 0.870, and the CR of total scale was 0.957, which is greater than the reference value of 0.6 and consistent with the standard. The AVE of preference for pop music was 0.711, which is greater than the reference value of 0.5 and consistent with the standard, thus indicating a good convergent validity (Anderson and Gerbing, 1988). Cronbach’s α of total scale was 0.957, which indicated good reliability.

### Common Method Variance

To test common method bias, Harman’s one-factor test is used (Harman, 1976). The eigenvalues of 5 factors in the present study are greater than 1. the first factor can only explain 45.398%, which is far less than the accumulated explanatory variance of 50%. Therefore, no serious common method bias problem exists in this study.

### Descriptive Statistics and Correlation Analysis

Table 3 shows that the correlation was significant and positive between pop music and mental health (*r* = 0.515, *p* < 0.001); between Western classical music and mental health (*r* = 0.453, *p* < 0.001); and between Chinese traditional music and mental health (*r* = 0.435, *p* < 0.001), whereas the correlation was significant and inverse between college students’ preference for heavy music and their mental health (*r* = −0.215, *p* < 0.001). Therefore, the correlation coefficients between all dimensions of college students’ music preference and their mental health were significant at the level of less than 0.8, indicating the absence of a strong correlation and thus of any serious collinearity.

**TABLE 3:** Summary of descriptive statistics and correlation analysis.

Variables	M	SD	Pop music	Western classical music	Chinese traditional music	Heavy music	Mental health
Pop music	3.589	1.299	1				
Western classical music	3.572	1.233	0.360***	1			
Chinese traditional music	3.724	1.188	0.428***	0.384***	1		
Heavy music	2.574	1.099	−0.022	−0.098	−0.116*	1	
Mental health	3.750	1.122	0.453***	0.435***	0.515***	−0.215***	1

*p < 0.05, ***p < 0.001.

## Discussion and Conclusion

This study showed that college students’ preference for popular, Western classical, and Chinese traditional music exhibited a significant positive correlation with their mental health. Similar findings have been reported in the literature. Popular music can help young people develop relationships as well as explore the personalities of others, provide a means for communication and common activities, and benefit their physical and mental health (Xu, 2021). Listening to Western classical music can help people release stress as well as relax their mind and body and contribute to their positive mental health (Rea et al., 2012). Chinese traditional music is characterized by nationality, inheritance, and regional characteristics, and students who listen to it may feel more familiar with it and relaxed (Liu et al., 2021).

In this study, college students’ preference for heavy music was found to have a significant and inverse correlation with their mental health. This finding is also consistent with those of some previous studies (Martin et al., 1993; Larson, 1995; Chen et al., 2006). Previous research has shown people who prefer heavy music often experience many psychological problems and are highly prone to develop negative emotions such as anger (Martin et al., 1993; Larson, 1995; Chen et al., 2006). Some researchers have reported that heavy music has correlation with the internalization problems of teenagers, such as depression, loneliness, anxiety, and stress (Bask, 2015; Schoemaker et al., 2019). However, the result of this research differs from that of some studies, which have reported that the preference for heavy music has a positive correlation with people’s mental health (Arnett, 1991; Quinn, 2019). This may be due to cultural differences between China and Western countries. Chinese college students are more conservative and introverted than Western college students who are open-minded and extroverted (Yang, 2012). Chinese college students lack the ability to release themselves (Zhou, 2005). A recent empirical study in China also found that Chinese college students tend to become vulnerable, lonely, and even irritable and present more psychological problems after listening to heavy music (Wang, 2021).

## Theoretical Contributions

This study focused on the relationship between music preference and mental health of Chinese college students. The results showed that the correlation between college students’ preference of pop, Western classical, as well as Chinese traditional music and mental health was significant and positive. By contrast, the results showed a inverse association between heavy music and mental health.

This study has made three major contributions. First, limited empirical studies have been conducted on the relation between music preference and mental health over the past years, and most of these studies were observational that focused on mental diseases and patients’ mental state by testing or conducting clinical trials (McFerran et al., 2018; Huang and Li, 2022). However, the present study involved a questionnaire survey for discussion and statistical analysis. Second, the music preference scales adopted in previous empirical studies were different owing to cultural differences between Chinese and Western countries (Schwartz and Fouts, 2003; Xu et al., 2010). However, the localized Chinese music preference scale adopted in this study helped us better understand the music preference of Chinese college students. Third, most of the previous studies were conducted in the Western context (Schwartz and Fouts, 2003), whereas the present study explored the correlation between Chinese college students’ music preference and their mental health by taking Chinese college students as the sample and in the context of Chinese culture. This study showed that the music preferences of college students for different music forms have different correlations with their mental health. The results of this study can provide evidence for further empirical studies on the correlation between music preference and mental health.

## Limitations and Future Research Directions

The following limitations of this study should be considered to interpret the findings. First, this study was based on cross-sectional data, and causality cannot be inferred. Future research should use a longitudinal design or cross-lagged panel analyses and apply an experimental approach to explore causality. Second, the study sample is college students selected using the convenience sampling method; therefore, the findings should be cautiously generalized to other samples. Finally, the study data are based on self-reports; hence, future research should collect qualitative data to understand the association of college students’ music preferences with mental health for a thorough analysis of the findings.

## Data Availability Statement

The raw data supporting the conclusions of this article will be made available by the authors, without undue reservation.

## Ethics Statement

The studies involving human participants were reviewed and approved by Graduate University of Mongolia. The patients/participants provided their written informed consent to participate in this study. Written informed consent was obtained from the individual(s) for the publication of any potentially identifiable images or data included in this article.

## Author Contributions

KW designed the study, collected and analyzed the data, and wrote the manuscript. SG and JH revised the manuscript. All authors contributed to the article and approved the submitted version.

## Conflict of Interest

The authors declare that the research was conducted in the absence of any commercial or financial relationships that could be construed as a potential conflict of interest.

## Publisher’s Note

All claims expressed in this article are solely those of the authors and do not necessarily represent those of their affiliated organizations, or those of the publisher, the editors and the reviewers. Any product that may be evaluated in this article, or claim that may be made by its manufacturer, is not guaranteed or endorsed by the publisher.
